# The tumor immune microenvironment: implications for cancer immunotherapy, treatment strategies, and monitoring approaches

**DOI:** 10.3389/fimmu.2025.1621812

**Published:** 2025-09-22

**Authors:** Kelsey Jane Racacho, Ya-Ping Shiau, Rodolfo Villa, Sohaib Mahri, Menghuan Tang, Tzu-Yin Lin, Yuanpei Li

**Affiliations:** ^1^ Department of Biochemistry and Molecular Medicine, University of California, Davis, Sacramento, CA, United States; ^2^ Department of Internal Medicine, University of California, Davis, Sacramento, CA, United States

**Keywords:** tumor immune microenvironment, immune surveillance, immune activation, immunosuppressive, treatment, artificial intelligence

## Abstract

The tumor immune microenvironment (TIME) plays a pivotal role in cancer progression, detection, and response to cancer treatments. Current knowledge of the diverse and dynamic cellular components of the TIME underscores how the immune landscape evolves in response to immunotherapy. This review highlights the importance of understanding the TIME for advancing cancer immunotherapy by integrating insights from basic biology and clinical practice with recent advances in science and technology, paving the way for more personalized cancer therapies through modern medical innovations. The cellular and molecular compositions of the TIME and the cellular interactions will be explored. Next, we summarize how the TIME is shaped by immune activation and suppression through various mechanisms of action. Immunotherapies designed to enhance host immune function are discussed in detail to visualize and quantify cellular dynamics within the TIME once treated with immunotherapy. In particular, the integration of artificial intelligence (AI) has significantly enhanced early cancer detection and diagnostics by analyzing patient samples with greater precision. The topics are structured to explore core principles, immune activation and suppression, imaging methods, current and emerging therapies, and the broader influence of the TIME on diagnosis, monitoring, and treatment strategies.

## 1 Introduction

The World Health Organization (WHO) has reported roughly 9.7 million cancer-related deaths around the world and nearly 20 million cancer-related incidences in 2022 (1). Globally, lung cancer was reported as the most common cancer type and the leading cause of cancer-related deaths in 2022. Among females, it was followed by breast cancer, colorectal cancer, prostate cancer, and stomach cancer, according to the WHO and the International Agency for Research on Cancer (2). Cancerous tumors arise from mutated cells that divide uncontrollably and can migrate to form new tumors—a process called metastasis that occurs in late-stage tumor development (3–5). Many cancer types form solid tumors, although some cancers, such as leukemias, form liquid tumors (6). Each tumor type has driver genes that promote tumorigenesis by supporting signaling pathways and creating a cancer-friendly environment, posing major challenges for treatment (7). Tumors create and sustain complex pro-tumorigenic environments called tumor immune microenvironments (TIME). TIME comprises host immune cells that can contribute to tumor progression by creating an immunosuppressive environment and facilitating tumor growth and expansion (8). TIME is a continuously evolving environment that best suits the specific tumor type, surrounding cells, and environment (8, 9). Due to the complexity of the TIME, monitoring and diagnostic tests have played an important role in cancer treatment to prevent progression and manage recurrence (10) We will discuss key cell types in the TIME - T lymphocytes (T cells), natural killer (NK cells), macrophages, and dendritic cells (DCs) - that help tumors evade the immune system and the complex interactions within the TIME presenting major challenges for effective cancer treatment (8). Current TIME-targeting treatments boost the immune system or directly attack tumor cells, using approaches like checkpoint inhibitors, adoptive cell therapy, and cancer vaccines (11, 12). Treating the TIME is challenged by its complexity, heterogeneity, and tumor immune evasion, leading to resistance and reduced efficacy, especially in solid tumors (13, 14). We will explore the complex immune landscape of the TIME, highlight key immune cell types and current immunotherapies, while addressing the challenges posed by tumor heterogeneity and immune evasion in cancer treatment.

### 1.1 Concepts of tumor immune microenvironment

TIME is a dynamic, multifaceted ecosystem composed of tumor cells, diverse immune populations—including tumor-infiltrating lymphocytes (TILs), macrophages, DCs, and myeloid-derived suppressor cells (MDSCs), as well as non-immune stromal components such as fibroblasts and endothelial cells, all of which work together to modulate anti-tumor immunity (15, 16). Tumor-host interactions shape the TIME as tumor-derived factors (tumor-derived cytokines, growth factors, and metabolites) promote tumor survival and remodel the microenvironment, while host immune and stromal cells provide nutrients and support that influence tumor progression (17, 18). Within the TIME, tumor cells mimic the host immune system to evade attacks and shape a specialized environment, while the host immune system continuously remodels the TIME through tumorigenesis and immune evasion, promoting chronic inflammation, a hallmark of cancer progression through the innate and adaptive immune response (8, 19, 20).

During immunosuppression, the immune system limits immune cell recruitment to the TIME by dampening the overall immune response (21). Immunotherapy can reshape the TIME by overcoming immune suppression and restoring the function of anti-tumor immune cells. The TIME is shaped by immunosuppressive cytokines, chemokines, and inflammatory growth factors, along with additional suppressive signals from lymphocytes, myeloid cells, macrophages, neutrophils, fibroblasts, and vascular-associated cells (18, 22, 23). The host immune system is responsible for identifying and neutralizing non-self-materials, such as foreign antigens. However, tumors can manipulate this system to shape the TIME by selectively promoting the survival of cancer cell variants that resist, evade, or suppress the anti-tumor immune response (20, 24). This host action can lead to the “escape phase,” which is mediated by immune cell types such as regulatory T cells (Tregs) and MDSCs that are known to inhibit anti-tumor cells (3, 25). This section of the review will summarize the components of the TIME that contribute to tumor progression, including extracellular matrix (ECM) remodeling, cell recruitment, pH and hypoxic selectivity, immune suppressive environment through cytokine signaling, and shifting immune metabolism.

### 1.2 Extracellular matrix

Within the TIME, tumor and non-cancerous cells, immune cells, blood vessels, fibroblasts, cytokines, and growth factors all interact with the ECM to drive tumor development and progression (8, 23). The ECM provides a physical barrier for the TIME by preventing the recruitment of host immune cells into the tumor and influencing immune cell activation. The ECM density and stiffness can prevent the movement of T cells and other host immune cells from making contact with tumor cells (26). The polarization of macrophages has been shown to be influenced by the ECM, initiating either the tumor-suppressive (M1) or tumor-promoting (M2) macrophages (27). The ECM can influence cells through cell adhesion molecules (CAMs), such as integrins, which bind to ECM components (e.g., collagen, fibronectin, and laminin). This interaction plays a crucial role in regulating cell adhesion, migration, and signaling (28). Specific CAMs, such as integrins, play a key role in ECM remodeling. Integrins activate various cellular processes through signaling pathways like focal adhesion kinase (FAK), phosphatidylinositol 3-kinase (PI3K), and Rho GTPases (e.g., Rho, Rac, and Cdc42). These pathways are involved in regulating cell motility, proliferation, migration, adhesion dynamics, cellular morphology, and cancer progression (29, 30). While cadherins are calcium-dependent adhesion molecules essential for mainly cell-cell interactions and ECM remodeling (31).

The ECM also contributes to the recruitment of MDSCs primarily through chemokine signaling, including the pathways mentioned above, thereby enhancing ECM-driven suppression of the host immune response (27, 32, 33). Targeting the ECM is promising due to its role in tumor progression. Tumor heterogeneity and signaling pathways in the TIME drive ECM remodeling, hypoxia, angiogenesis, and immune evasion complicating therapies and promoting tumor growth and resistance (34).

### 1.3 Hypoxia

The high nutrient consumption in the TIME creates hypoxic conditions, leading tumors to shift their metabolism toward elevated glycolysis as their primary energy source, even in the presence of oxygen, a phenomenon known as the Warburg effect (35, 36). Rapid tumor growth and metabolism produce acidic byproducts like lactate and CO_2_, creating toxic metabolites (ex. lactate, oxidized lipids, and adenosine) that suppress host immunity and promote tumor progression (37, 38). In the hypoxic conditions of the TIME, immune cell recruitment, the accumulation of immunosuppressive cells, and the upregulation of immunosuppressive molecules all contribute to tumor progression (39). Hypoxia-inducible factors (HIFs) are transcription factors that regulate tumor cell responses in a hypoxic environment by activating transcription of genes involved in tumor cell proliferation, angiogenesis, metabolism, and invasion (40, 41). Common HIFs are HIF-1α (HIF1A) and HIF-2α (HIF2A), both of which play key roles in tumor growth, and metastasis (42).

### 1.4 Abnormal tumor vasculature

One of the defining features of the TIME is its abnormal vasculature, which results from the inadequate oxygen supply to the tumor site (43). Once a tumor grows beyond a few millimeters, it releases growth factors, directing tumor and non-cancerous cells to grow, divide, differentiate, or undergo cell death (44). The tumor compensates for oxygen deprivation by inducing angiogenesis, a process of new blood vessel formation in response to increased oxygen demand (45), which is critical for tumor growth and vascularization (46, 47). The abnormal structure of blood vessels is often referred to as tumor vasculature and is associated with leaky barriers and cancer progression (48, 49). Disorganized vasculature hinders treatment delivery due to reduced blood flow throughout the tumor site and elevated fluid pressure, leading to uneven treatment distribution, creating a physical barrier for treatments, and impacting treatment efficacy (50). Irregular vasculature, acidosis, and high pressure hinder T-cell infiltration, promoting immune escape (49). Angiogenesis is also known to help regulate TIME by providing oxygen and manipulating various host cells, immune cells and nutrients using the irregular vasculature network to evade immune surveillance (51–54).

### 1.5 Tumor acidosis and immune modulation

The complexity of the TIME relies only on the increased use of host physiological supplies and nutrients and a tightly regulated pH of 6.7-7.1 and, specifically, a pH below 7.2 within the tumor site (55–57). The acidic environment suppresses and disrupts the host immune response by impairing their ability to proliferate, migrate, and produce cytokines (58–60). Acidity, specifically, lactic acid buildup in the TIME in an aerobic and anaerobic environment impairs lymphocytes and macrophages, especially during inflammation (61, 62). Acidosis impairs T cell function by reducing the production of key cytokines such as interferon-gamma (IFN-γ) and tumor necrosis factor-alpha (TNF-α), as well as cytotoxic molecules like perforin and granzyme. An acidic extracellular environment can influence cytotoxicity by modifying cell cycle kinetics, inhibiting cell death, and impairing cytotoxic functions (63, 64). Acidosis can significantly affect immune cell function by inhibiting DC maturation and altering macrophage polarization, specifically promoting M2 polarization, which can contribute to immunosuppression within the TIME (65, 66). A preclinical study showed that an acidic TIME promotes local invasion and metastasis in breast, colorectal, and colon cancers, supporting acid-mediated tumor progression in a colorectal mouse model (66, 67). The change in acidity stems from tumor cell metabolism increases acid production, lowering pH and creating a toxic environment that impairs immune cells, chemokines, and cytokines (60).

### 1.6 Immunosuppressive role of cytokines

Immune tolerance is maintained by suppressive cytokines that limit inflammation and prevent immune attacks, but tumor cells exploit this mechanism by reducing pro-inflammatory signals and weakening anti-tumor immunity (68, 69). However, dysregulation of immunosuppressive cytokines can lead to excessive or prolonged production of immunosuppressive cytokines, resulting in immunosuppression of the host (21). The immunosuppressive cytokine interleukin-10 (IL-10), produced by macrophages, monocytes, and T cells, can inhibit the production of pro-inflammatory cytokines and suppress the activation of T cells and NK cells (70). The accumulation of IL-10 can reduce the function of antigen-presenting cells, lowering the adaptive immune response (71). Another key cytokine is Interleukin-35 (IL-35), produced by regulatory T cells, inhibits the proliferation and function of effector T cells while promoting the development of immunosuppressive microenvironments (72). IL-35 inhibits pro-inflammatory cells and its cytokines while enhancing the production of anti-inflammatory cytokines such as IL-10 and transforming growth factor-beta (TGF-β). TGF-β, in turn, suppresses T cell proliferation and activation, reduces inflammatory cytokine production, and promotes regulatory T cell differentiation (73). IL-10 and IL-35 play critical roles in the development of TIME due to their significant role in modulating the immune response, suppression of anti-tumor immunity and the promotion of tumor growth (74). Cytokines inhibit immune function in immunosuppression by regulating cell activation, differentiation, and metabolism (75).

Immune metabolism is a critical component that reflects the metabolism effects on the immune response, overall impacting tumor growth and development (76). Specifically, cytokines influence the activity of immune cells and also impact the metabolism by tailoring the energy and nutrients the cells use (77). The activation, differentiation, and function of immune cells are dependent on energy supply and metabolic transformation signaling (78). Tumor-intrinsic signaling fosters the exclusion and dysfunction of effective immune cells. Specifically, oncogenic drivers, including β-catenin, signal transducer and activator of transcription 3 (STAT3), phosphatidylinositol 3-kinase (PI3K), phosphatase and tensin homolog (PTEN), protein kinase B (AKT), mechanistic target of rapamycin (mTOR) (PI3K/PTEN/AKT/mTOR), p53, nuclear factor kappa-light-chain-enhancer of activated B cells (NF-κB), and Ras/Raf/mitogen-activated protein kinase (RAS/RAF/MAPK) signaling, are activated within the TIME and are critical for tumor development and metabolism (79). These oncogenic pathways reduce chemokine production, further hindering the recruitment of DCs, macrophages, T cells, and NK cells to the tumor site and promote the immunosuppression of host immune cells. Tumor-intrinsic signaling induces programmed cell death ligand 1 (PD-L1) expression, promoting T cell dysfunction and immune suppression, hindering tumor control and treatment (80).

## 2 Critical players for activating the immune system and suppressing tumor formation

**Figure 1:**
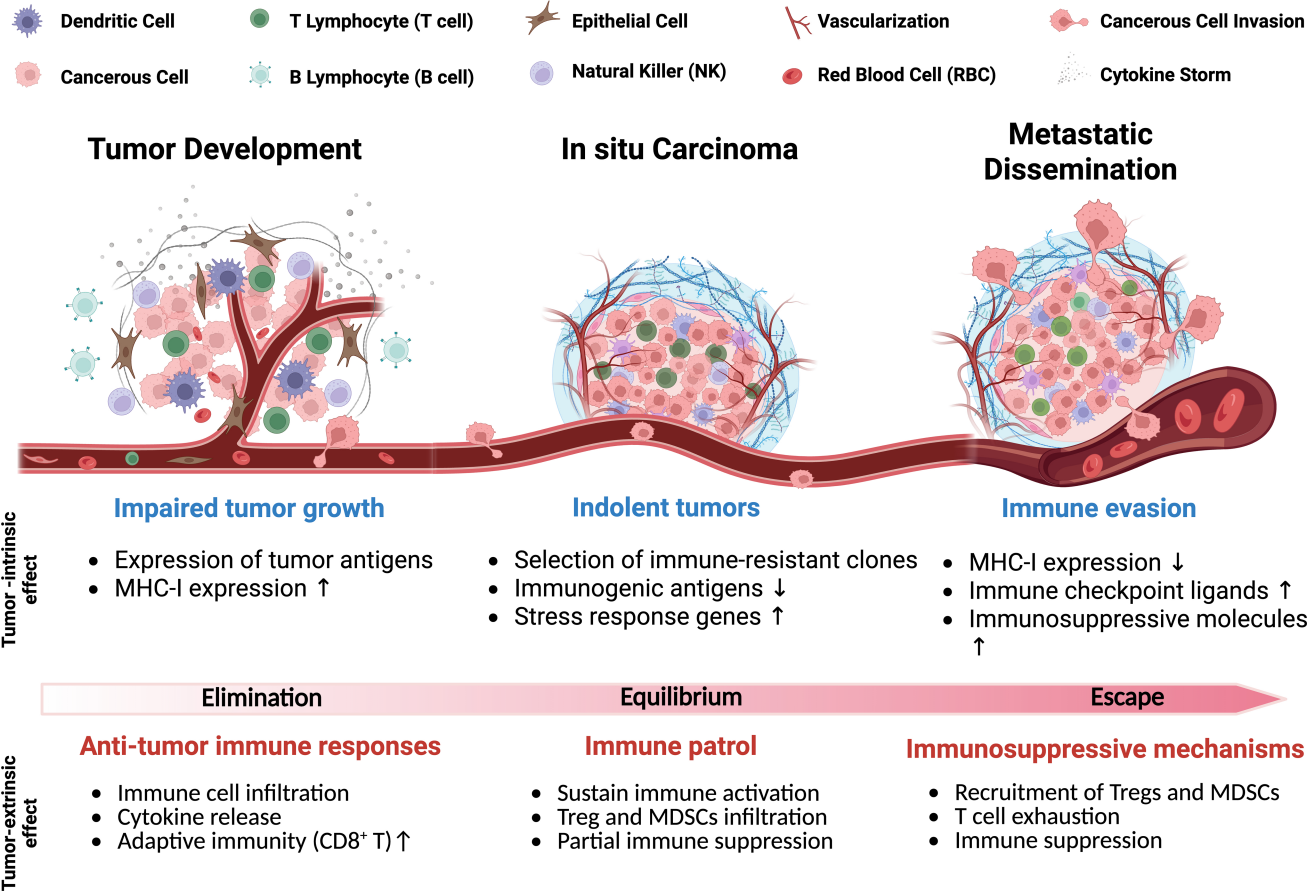
Schematic of tumor progression highlighting key physiological features and cell types. The gradient illustrates intrinsic and extrinsic factors driving the shift from anti-tumor immunity to immune evasion, including elimination and escape mechanisms used by tumors to suppress host immune responses.

The TIME is made up of diverse cell populations consisting of immune cells, cancerous and non-cancerous cells, each requiring optimal conditions to perform its specific function, collectively influencing tumor progression, **Figure 1** (9). In early tumor development, dendritic cells (DCs) and macrophages recognize tumor-associated antigens and initiate inflammation by releasing pro-inflammatory cytokines (e.g., IL-6, TNF-α, IL-1β) and chemokines (e.g., CXCL2, CXCL9, CXCL10), which recruit immune cells to the tumor site to help prevent progression (81, 82). Additionally, DCs and macrophages both are antigen-presenting cells (APCs), which process and present tumor antigens through MHC-I molecules by stimulating the proliferation of CD8^+^ cytotoxic T cells for further tumor cell elimination (83, 84). In response, tumor cells experience immunological stress that temporarily slows their proliferation due to increased antigen expression and immune-mediated cytotoxicity (24). However, tumor cells adapt and evolve new mechanisms to evade immune detection by selecting immune-resistant clones, downregulating highly immunogenic antigens, and upregulating stress response genes, such as HIF-1α and STAT3, to compensate for host immune response (85, 86).

**Figure 2:**
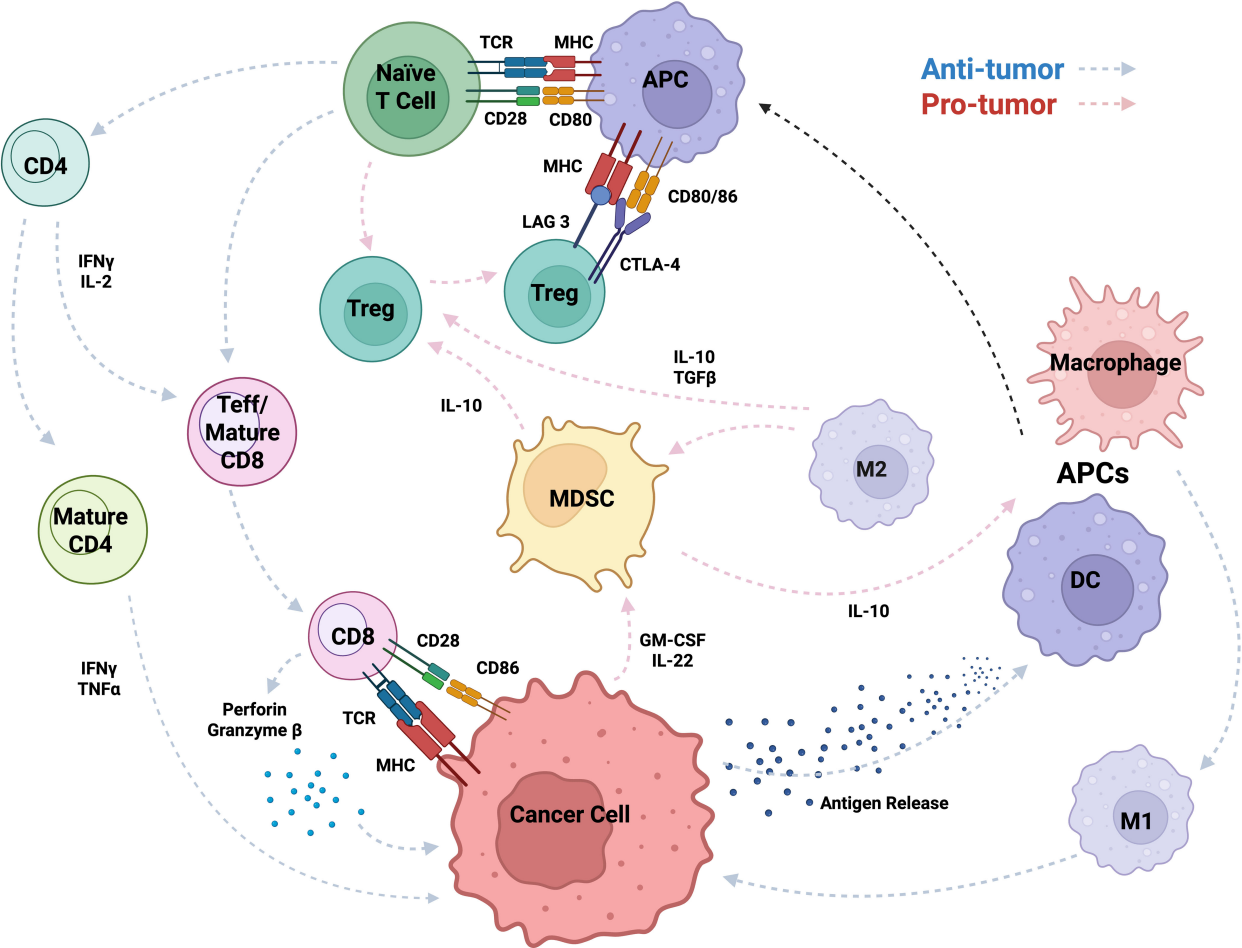
Immune cell crosstalk and regulatory mechanisms within the TIME, highlighting key cytokine signaling pathways and cellular functions. The schematic illustrates interactions among immune cells (e.g., T cells, APCs, MDSCs, macrophages, DCs, TAMs, Tregs, Teffs) through cytokines (e.g., IL-2/10/22, GM-CSF, TGF-β), molecules (e.g., perforin, granzyme B, IFN-γ), and receptors/ligands (e.g., TCR, MHC, CD28, CD80/86, LAG-3, CTLA-4), showing how signaling drives activation or suppression in the tumor microenvironment.

Stress response genes help tumor cells resist immune-induced apoptosis and survive inflammation by supporting protein refolding and degradation contributing to tumor cell survival from the hosts’ immune system (87). Stress response genes can suppress the host immune system, impairing its ability to attack TIME. In turn, the TIME recruits MDSCs and Tregs, which further inhibit immune responses, promote immune evasion, and support tumor progression (88, 89). The immunosuppressive cells dampen immune activation, creating a tolerant microenvironment that weakens the host anti-tumor response (90). In the late stages of tumor development, the host immune system becomes progressively ineffective as tumor cells upregulate immune checkpoint ligands, such as PD-L1 and cytotoxic T lymphocyte-associated protein 4 (CTLA-4) (89, 91), which inhibit CD8^+^ T cell-mediated cytotoxicity (21). Continuous activation of Tregs further suppresses effector T cell responses, which can reinforce an immune-tolerant state of the TIME (92). Under these conditions, tumor cells are primed for metastasis, acquiring the ability to invade surrounding tissues, migrate through the bloodstream, and colonize distant organs (92, 93). Here, we highlight key tumor-associated immune cells and their roles in modulating cancer progression **Figure 2** and **Table 1**.

**Table 1:** Key cell types and receptors in the TIME and their roles.

Immune cells		Canonical Function	Anti-tumor Mechanisms	Pro-tumor Mechanisms	Targeted Therapeutic Strategies	Ref
Neutrophil (CD15)		• Innate responder to infection and inflammation.	• Tumor killing via ROS, MMP-9, H_2_O_2_, TRAIL, FasL/Fas, NETs, ADCC and cytokine production.	• Promotes angiogenesis (VEGF). • ECM remodeling. • Immunosuppression (ARG1). • Metastasis via NETs.	• Blocks CXCLR1/2 and C5aR to reduce neutrophil suppression. • NETosis inhibitors.	(94–98)
NK cells (CD56)		• Innate cytotoxic lymphocytes. • Regulate tumors and infections. • Control cell proliferation and limit tissue damage.	• Cytotoxicity via IFN-γ and perforin/granzyme, ADCC. • Promotes cell killing of MHC-presenting cells without prior sensitization.	• Dysfunction in TME due to hypoxia, TGF-β. • Reduced cytotoxicity and infiltration.	• CAR-NK therapies • Target exhaustion-related receptors (e.g., PD-1, TIM-3). • Modulate NK cell state to restore tumor-killing activity	(99–105)
T cells (CD3) (CD4^+^ Th) (CD8^+^ Tc)		• Adaptive immunity effects in both anti-tumor response and TIME protection.	• CD8^+^ Tc cells suppress tumor growth and enhance host immune response. • CD4^+^ Th1 cells enhance CTL and DC function.	• Treg cells promote tumor progression and metastasis. • Exhausted T cells lose effector function.	• ICIs (e.g., anti-CTLA4). • CAR-T cell therapy. • High T-cell infiltration, such as Fc-enhanced anti-CTLA4 antibodies (BMS-986218 and ONC-392). • Blocking other Treg markers along with CTLA4, such as CCR7 and TIGIT.	(106–111)
MDSCs (CD11b^+^Gr1^+^)		• Promotes immune evasion	• (minimal role) Potential antigen presentation	• Inhibit T cell, DCs and NK cells function via oxidative stress. • Promote Tregs, M2 macrophages.	• MDSC depletion, such as anti Gr-1 antibodies, CXCR1/2 or Src inhibitors and chemotherapy. • Suppress function using PDE5 and Galectin-3 inhibitors.	(25, 112–116)
APCs	DCs (CD11c)	• Antigen presentation via MHC I/II	• Activate CD4^+^/CD8^+^ T cells,by producing IL-12, TNF-α, IL-6.	• Upregulate PD-L1, IL-10, and TGF-β. • Promoting Treg expansion	• STING agonists. • DC vaccines. • Expand DCs (e.g., Flt3L and CHNPs).	(84, 117–120)
	Macrophage (CD68 M1) (CD163 M2)	• Phagocytosis • Both anti-tumor (M1-like) and pro-tumor (M2-like, TAM).	• M1-like macrophages support immune responses by producing IL12, TNF-α.	• M2-like TAMs promote angiogenesis, immune suppression, tissue remodeling, Treg expansion, and inhibit cytotoxic T cells.	• Reprogramming TAMs to M1, via CD40/TLR agonists. • TAM depletion via CSF1R inhibitors (e.g., Pexidartinib) and CCR2 inhibitors (e.g., PF-04136309). • CD47-SIRPα blockade enhances phagocytosis.	(121–125)

ADCC, antibody-dependent cellular cytotoxicity; ARG1, arginase 1; ATRA, all-trans retinoic acid; APCs, antigen presenting cells; CAR-NK, chimeric antigen receptor-natural killer cells; CAR-T, chimeric antigen receptor T cells; CHNP, cytotoxic hybrid neutrophil population; CSF1R, colony-stimulating factor 1 receptor; CTL, cytotoxic T lymphocyte; DCs, dendritic cells; ECM, extracellular matrix; Flt3L, fms-like tyrosine kinase 3 ligand; MDSC, myeloid-derived suppressor cell; NETs, neutrophil extracellular traps; NK cells, natural killer cells; PDE5, phosphodiesterase type 5; ROS, reactive oxygen species; STING, stimulator of interferon genes; TAMs, tumor-associated macrophages; TIM3, T cell immunoglobulin and mucin domain-containing protein 3; TME, tumor microenvironment; TRAIL, TNF-related apoptosis-inducing ligand; Treg, regulatory T cells; VEGF, vascular endothelial growth factor; VISTA, V-domain Ig suppressor of T cell activation.

## 3 Characterizing immune activation within the TIME

The tumor immune microenvironment (TIME) is a dynamic ecosystem where cancer cells, stromal cells, cancer-associated fibroblasts (CAFs), endothelial cells, and immune cells (including the extracellular matrix, ECM) engage in multifaceted interactions. These interactions contribute to tumor development, progression, and protection, ultimately shaping the tumor’s fate and response to therapies (126, 127). Due to its modifiable immunosuppressive environment and potential to enhance immunotherapy effectiveness, TIME is a promising therapeutic target (128).

RNA sequencing and immunohistochemistry from the ORIENT-11 study to optimize a TIME classification model predicting non-small cell lung cancer (NSCLC) treatment outcomes. The TIME is classified into four types based on PD-L1 and TIL status. Patients with high PD-L1 and TIL benefit from combined chemo-immunotherapy, while those with low levels respond better to chemotherapy alone to avoid unnecessary toxicity. However, this model’s efficacy lacks validation in large-scale randomized trials (129).

**Figure 3:**
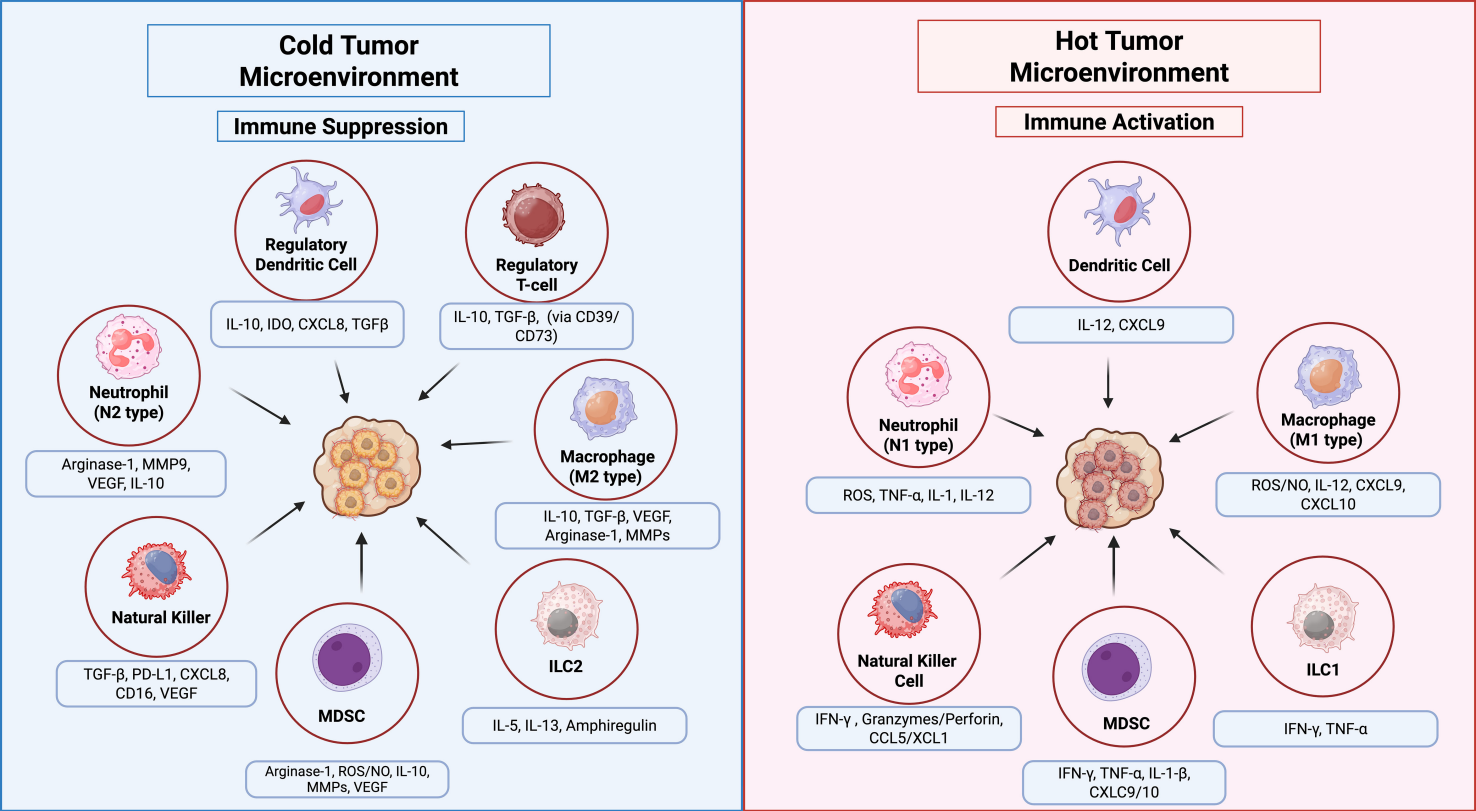
Innate immune cell functions in the TIME of hot vs. cold tumors. In hot tumors (right panel), high immune infiltration supports anti-tumor responses via NK cells, M1 macrophages, ILC1s, and N1 neutrophils through pro-inflammatory cytokines. In cold tumors (left panel), low immune infiltration and immunosuppressive cells, M2 macrophages, ILC2s, regulatory DCs, N2 neutrophils, MDSCs, and Tregs promote tumor growth. Arrows indicate key cellular interactions and cytokine effects.

Additionally, tumor-associated immune cells can be classified into two categories: anti-tumor and tumor-promoting populations. Anti-tumor immune cells primarily include effector T cells (such as cytotoxic CD8^+^ T cells and effector CD4^+^ T cells), NK cells, DCs, and M1-polarized macrophages (127, 130). In contrast, anti-tumor immune cells are predominantly composed of Tregs, MDSCs, M2-polarized macrophages, N2-polarized neutrophils, type 2 natural killer T cells (NKT2), and innate lymphoid cells type 2 (ILC2s) (131). **Figure 3** illustrates the dual roles of immune cells in the TIME, highlighting pro-tumoral and anti-tumoral functions in hot and cold tumors with key cellular interactions and cytokine effects. Furthermore, various metabolic and biochemical components within the tumor microenvironment significantly influence immune cell function (108).

Immunotherapy-induced changes in the TIME offer insights for improving combination treatments and patient outcomes. Targeting the TIME reveals cancer-specific responses and aids in detecting development and recurrence. Immune activation occurs when immune cells recognize tumor antigens, triggering responses against both tumor cells and the TIME (9). Targeted immunotherapies can utilize not only cell-based treatments but also immune-modulating treatments. These latter treatments consist of nanoparticles decorated with cell-specific ligands, antibodies, or peptides designed to actively bind to the cell surface of targeted cells. In combination with other therapies, nanoplatforms interact with the TIME, modulating tumor associated macrophages (TAMs) and Tregs, and directly activates immune cells, enhancing antigen presentation, and promoting immunological memory formation (132). Antibody-functionalized nanoparticles enable precise targeting. Such as, monoclonal antibody-coated magnetic nanoparticles (MgNPs) loaded with doxorubicin showed 1.8-fold greater accumulation in Met/HGFR-positive tumors than non-functionalized MgNPs, enhancing drug retention, reducing toxicity and demonstrating strong potential for targeted delivery (133).

### 3.1 Activation of the immune system

Immune activation overcomes the tumor’s immunosuppressive microenvironment, transforming the TIME from resistant to responsive to cancer treatment by modifying its cellular and molecular composition. This enhances the host immune system’s ability to recognize and attack tumors, boosting T cell-mediated destruction, oncolytic viruses, cancer vaccines, and combination therapies (108). One critical route of cell death is immunogenic cell death (ICD), which is maintained and activated by the host’s immune system. ICD is a form of cell death initiated by certain chemotherapeutic drugs, oncolytic viruses, physicochemical therapies, photodynamic therapy, and radiotherapies (134, 135). When ICD occurs in cancer, it promotes dying cells secreting damage-associated molecular patterns (DAMPs), which act as alarm signals that activate the immune system against tumor cells. This process effectively turns dying cancer cells into natural “vaccines,” enhancing anti-tumor immunity. Key DAMPs such as surface-exposed calreticulin, secrete ATP, and the nuclear protein high-mobility group box 1 (HMGB1), increases tumor immunogenicity and attracts immune cells to the TIME (134, 136, 137). ICDs have mediated patterns that reveal immune microenvironment classification and immunotherapeutic responses in bladder cancer and many other cancer types. Fu et al., 2022, revealed a novel paradigm for characterizing the immune microenvironment and immunotherapeutic responses in bladder cancer, identifying two patterns of TIME and responses to immunotherapy of ICD within bladder cancer. The two patterns include an ‘immune-inflamed’ type, characterized by abundant activated immune cells and favorable immunotherapy response, and an ‘immune-excluded’ type, where immune cells are limited or localized to the tumor’s outer regions, associated with less favorable outcomes. These TIME patterns help predict which patients are more likely to benefit from immunotherapeutic strategies like immune checkpoint inhibitors.

The main characterization found distinct clinical features of the TIME, exhibiting unique clinical and immune characteristics of an ICD cluster, despite being linked to a poor prognosis and high ICD score, demonstrated an immune-activated state. This activation was associated with a more favorable response to immunotherapy and ICD-inducing treatments leading to further investigation in ICD-related gene, *CALR*. *CALR* was significantly overexpressed in the T24 bladder cancer cell line compared to the control SV-HUC-1 cells. Experimental knockdown of *CALR* reduced T24 cell viability and triggered endoplasmic reticulum stress, highlighting its potential role in cancer cell survival, stress response, and activation of the immune system (138).

Chemotherapy induces ICD by exposing calreticulin, releasing ATP, and secreting HMGB1, which signals immune cells to recognize dying cancer cells (135). Treating NSCLC cells with 10 µM Crizotinib, an ICD-inducing tyrosine kinase inhibitor, enhances anticancer effects when combined with non-ICD chemotherapy like Cisplatin. This combination boosts tumor T cell infiltration and PD-1/PD-L1 expression, improving response to PD-1 immunotherapy. Removing T cells or blocking interferon-γ negates these effects, highlighting a promising strategy of chemotherapy plus Crizotinib followed by immune checkpoint inhibitors for NSCLC (139). One study compared the treatment method of preoperative neoadjuvant chemoradiotherapy (nCRT), a treatment using chemotherapy and radiation therapy before surgery to shrink a tumor (140). nCRT is used to potentially remove or increase the likelihood of a less invasive surgery which is the primary therapeutic strategy for patients with locally advanced rectal cancer (LARC). A Systematic Review and Meta-analysis revealed nCRT was associated with improved pathologic complete response rates and has a potential disease-free survival advantage compared to the standard CRT plus (140, 141). Huang et al. found that using a novel topoisomerase I inhibitor, Lipotecan (TLC388), induces ICD, leading to the release of damage-associated molecular patterns (DAMPs) such as HMGB1, ANXA1, and exposure of calreticulin. This process increases cancer immunogenicity and stimulates the host’s anti-tumor response, promoting immune cell infiltration in the TIME of colorectal cancer. These findings suggest Lipotecan can reshape the TIME to boost neoCRT effectiveness in LARC patients (142).

### 3.2 Innate immune recruitment and activation in the TIME

In a healthy system, macrophages are generally of the M2-like subtype and produce anti-inflammatory cytokines such as IL-10 and TGF-β, which contribute to inflammation resolution and tissue regeneration (143, 144). Neutrophils also control tissue homeostasis by producing lipid mediators such as resolvins and lipoxins that tune the immune response (145, 146). Macrophages and neutrophils help maintain immune homeostasis through efferocytosis, the process of clearing apoptotic cells and cellular debris, promoting a controlled immune response essential for tissue repair and normal physiological function (147, 148). Moreover, both cells exhibit functional plasticity in the TIME by either promoting or suppressing tumors. Activated macrophages polarize into M1 and release pro-inflammatory cytokines (e.g. TNF-α and IL-12) and ROS to activate T cells and trigger tumor cell death (149). As tumors progress, the TIME often shifts to favor M2-like TAMs that secrete immunosuppressive factors such as IL-10 and TGF-β and suppress immune responses and support tumor growth (149). Similarly to macrophages, neutrophils in the TIME show plasticity, adopting anti-tumor (N1) or pro-tumor (N2) states based on cytokine signals. N1 neutrophils support anti-tumor immunity by releasing pro-inflammatory cytokines, activating T cells, and recruiting CD8^+^ T cells to kill tumor cells (150). N2 neutrophils contribute to angiogenesis, immune suppression, and metastasis by the production of VEGF, arginase-1, and MMP9 (151). Neutrophils can influence almost every aspect of cancer progress, primary tumor growth, metastasis, cancer stem cell maintenance and therapeutic resistance (150). Neutrophils recruit macrophages and DCs via chemokines, creating a feedback loop that amplifies immune activation (152). Due to the important participation of innate immunity in TIME, it also influences dynamic neutrophil polarization. Specifically, TGF-β promotes N2 differentiation, while type I interferons or TGF-β blockade shift polarization toward the anti-tumor N1 phenotype (150, 152, 153).

### 3.3 Modulation of immune checkpoint activation

Immune checkpoints such as PD-1/PD-L1 and CTLA-4 help maintain immune homeostasis by balancing activating and inhibitory signals to prevent excessive immune responses. PD-1, expressed on activated T cells, binds to PD-L1 on antigen-presenting or parenchymal cells, delivering inhibitory signals that suppress T cell proliferation, cytokine production (e.g., IFN-γ, IL-2), and cytotoxic activity (154). This interaction ensures autoreactive T cells do not attack healthy tissues by inducing functional exhaustion or apoptosis of self-reactive clones. Similarly, CTLA-4 competes with the costimulatory receptor CD28 for the ligands CD80/CD86, increases the naive T cell activation threshold and promotes Treg suppressive functions (155). These checkpoints also enforce peripheral tolerance through dual mechanism where PD-1/PD-L1 interactions directly inhibit effector T cell responses in tissues, whereas CTLA-4 modulates early T cell priming in lymph nodes and amplifies Treg-mediated suppression (91). In contrast to healthy cells, which dynamically regulate PD-L1 expression during inflammation to prevent collateral damage, the TIME hijacks this pathway by persistently overexpressing PD-L1 to evade immune responses. This dysregulation disrupts immune balance and promotes immune escape (156).

Immune checkpoint inhibitors (ICIs), for example, anti-PD-1/PD-L1 and anti-CTLA-4 antibodies, have revolutionized cancer therapy by restoring the function of exhausted T cells and renewing anti-tumor immunity. However, their efficacy varies widely across tumor types and is influenced by TIME factors such as T cell infiltration density, PD-L1 expression levels, and tertiary lymphoid structures (131). Despite their recent clinical success, resistance to ICIs remains a major challenge, frequently arising via compensatory upregulation of alternative inhibitory checkpoints (such as LAG-3 and TIM-3) and persistent epigenetic reprogramming of T cells within the TIME. Strategies to overcome this resistance include dual checkpoint blockade (e.g., targeting both PD-1 and LAG-3; see **Table 2**), as well as combining ICIs with therapies that target immunosuppressive components of the TIME, such as CSF-1R inhibitors that deplete tumor-associated macrophages (157).

**Table 2:** Approved and emerging immune checkpoint inhibitors.

Approved Immune checkpoint inhibitors								
Drug	Target	Cells		Type	Approved indications			
Atezolizumab (Tecentriq)	PD-L1	Tumor, APCs		mAb	NSCLC, SCLC, HCC, melanoma, breast cancer, urothelial cancer			
Avelumab (Bavencio)	PD-L1	Tumor, APCs		mAb	Merkel cell carcinoma, urothelial carcinoma, renal cell carcinoma			
Durvalumab (Imfinzi)	PD-L1	Tumor, APCs		mAb	NSCLC, urothelial carcinoma			
Nivolumab (Opdivo)	PD-1	T cells		mAb	Melanoma, NSCLC, SCLC, HCC, HL, renal cell carcinoma, HNSCC, urothelial carcinoma, MSI-H/dMMR colorectal cancer.			
Pembrolizumab (Keytruda)	PD-1	T cells		mAb	NSCLC, SCLC, HNSCC, HL, melanoma, urothelial carcinoma, MSI-H/dMMR CRC, MSI-H/dMMR cancers, gastric cancer, cervical cancer, HCC, Merkel cell carcinoma, renal cell carcinoma, esophageal carcinoma, endometrial cancer			
Cemiplimab (Libtayo)	PD-1	T cells		mAb	Cutaneous squamous cell carcinoma (CSCC), NSCLC, basal cell carcinoma (BCC).			
Nivolumab (Opdivo)	PD-1	T cells (CD8+)		mAb	Melanoma, NSCLC, SCLC, HCC, HL, renal cell carcinoma, HNSCC, urothelial carcinoma, MSI-H/dMMR colorectal cancer.			
Toripalimab (Loqtorzi)	PD-1	T cells		mAb	Nasopharyngeal carcinoma, Melanoma.			
Sintilimab (Tyvyt)	PD-1	T cells		mAb	HL, non-squamous and squamous NSCLC, HCC, esophageal squamous cell carcinoma, and gastric cancer.			
Camrelizumab (AiRuiKa)	PD-1	T cells		mAb	HCC, HL, esophageal squamous cell carcinoma, and nasopharyngeal carcinoma.			
Tislelizumab (Tevimbra)	PD-1	T cells		mAb	Esophageal squamous cell carcinoma, NSCLC, Gastric or gastroesophageal junction adenocarcinoma, HL.			
Retifanlimab-dlwr (Zynyz)	PD-1	T cells		mAb	Merkel cell carcinoma.			
Ipilimumab (Yervoy)	CTLA-4	Treg cells		mAb	Melanoma, metastatic, renal cell carcinoma, MSI-H/dMMR CRC.			
Tremelimumab (Imjudo)	CTLA-4	T cells		mAb	Unresectable HCC, NSCLC.			
Relatlimab +Nivolu mab (Opdualag)	LAG-3	Exhausted T cells		mAB	Melanoma.			
Immune checkpoint inhibitors in advanced clinical trials								
Drug	Target (T cells)		Type		Combination	Indications	Phase	Reference
Favezelimab	LAG-3		mAb		Pembrolizumab/lenvatinib Egorafenib, TAS-102 Pembrolizumab Pembrolizumab Pembrolizumab.	cSCC, Endometrial Cancer CRC CRC cHL cHL,DLBCL,iNHL.	3 3 3 2/3	NCT06036836 NCT05600309 NCT05064059 NCT05508867 NCT03598608
Eftilagimod alpha (efti)	LAG-3		Soluble LAG-3 fusion protein.		Paclitaxel Pembrolizumab.	Breast cancer cHL, DLBCL,iNH.L.	2/3 1/2	NCT05747794 NCT03598608
Tebotelimab	PD-1 & LAG-3		Bispecific mAb.		Margetuximab/Chemotherapy	HER2+GC or GEJ.	2/3	NCT04082364
Fianlimab	LAG-3		mAb.		Cemiplimab/Chemotherapy (Pemetrexed, Paclitaxel, Carboplatin, Cisplatin) Cemiplimab Cemiplimab.	NSCLC Metastatic melanoma Metastatic Melanoma Melanoma.	2/3 3	NCT05785767/NCT05800015 NCT05352672 NCT06246916 NCT05608291 NCT06190951
Botensilimab + Balstilimab	LAG-3 + PD-1		mAbs		- -	MSI/​dMMR Esogastric Adenocarcinomas (CIME) dMMR and pMMR solid tumors	3 3	NCT06346197 NCT06279130
Bispecific T-Cell Engagers (BiTEs)								
Drug	Target		Type		Indications			Year
Blinatumomab (Blincyto)	CD19 x CD3		BITE		r/r B-cell precursor ALL, MRD-positive B-ALL			2014
Mosunetuzumab (Lunsumio)	CD20 x CD3		BITE		FL			2022
Teclistamab (Tecvayli)	BCMA X CD3		BITE		MM			2022
Glofitamab (Columvi)	CD20 x CD3		BITE		DLBCL			2023
Epcoritamab (Epkinly)	CD20 × CD3		BITE		DLBCL			2023
Talquetamab (Talvey)	GPRC5D × CD3		BITE		MM.			2023
Tebentafusp (Kimmtrak)	gp100 x CD3		BITE		Metastatic uveal melanoma.			2022
Tarlatamab (Imdelltra)	DLL3 x CD3		BITE		Extensive-stage SCLC.			2024

cHL, classical Hodgkin lymphoma; CRC, Colorectal Cancer; cSCC, Cutaneous Squamous Cell Carcinoma; DLBCL, diffuse large B-cell lymphoma; dMMR, Mismatch Repair Deficient; GC, Gastric cancer; GEJ, Gastroesophageal Junction; HCC, Hepatocellular Carcinoma; HL, Hodgkin’s Lymphoma; HNSCC, Head and Neck Squamous Cell Carcinoma; iNHL, indolent non-Hodgkin lymphoma; LAG-3, Lymphocyte Activation Gene-3; mAb, monoclonal antibody; MSI-H, Microsatellite Instability-High; NSCLC, Non-Small Cell Lung Cancer; P, phase; pMMR, proficient mismatch repair; SCLC, Small Cell Lung Cancer.

DCs, key antigen-presenting cells, process tumor antigens via MHC and activate adaptive immunity by detecting pathogens through pattern recognition receptors (PRRs) like Toll-like receptors (TLRs) (158). The DC takes up the pathogen and then displays the antigens to T cells on MHC molecules, resulting in an immune response (159, 160). Activated DCs express moderate levels of costimulatory molecules like CD80 and CD86, enabling effective T cell activation, clonal expansion, and memory formation (161, 162). This maturation process enables T cells to receive the required signals for clonal expansion and the production of potent memory responses. Adaptive immune responses involve tumor-specific neoantigens that are released during immunogenic cell death (ICD). Processes involving DAMPs such as calreticulin, ATP, and HMGB1, also activate dendritic cells (DCs) and T cell priming to enhance anti-tumor immunity (163). This process allows for the recruitment and expansion of polyclonal cytotoxic and memory T cells and the establishment of systemic tumor-specific immunity and long-term immunological memory. In addition, DCs may also possess suppressive properties by inducing the activation of Tregs instead of effector T cells (164).

## 4 Characterizing immune suppression within the TIME

Tumors can evade the host immune system by creating an immunosuppressive microenvironment (165) One major mechanism involves impaired antigen presentation, typically through the downregulation of MHC molecules or other antigen-presenting proteins, which disrupts T cell recognition and weakens the antitumor immune response (166). Tumor cells may also mask their antigens with protective molecules, further preventing immune recognition, while the TIME releases factors that inhibit immune cells, such as, T cells, NK cells, and dendritic cells which promote immune tolerance and drive an unresponsive, immunosuppressive state (166, 167). Additionally, Tumor cells recruit immunosuppressive regulatory cells such as Tregs and exploit immune checkpoint pathways to suppress effector immune cell function. Cancer cells also develop cytoprotective mechanisms, like resistance to apoptosis, allowing them to evade immune-mediated death and modify the microenvironment to support their survival, growth, and continued immune evasion (68, 168). T cell exhaustion can also occur, leading to immune cell dysfunction and an inability to effectively target the tumor (169). To counteract these suppressive mechanisms, various clinical strategies have been developed to activate the TIME. These include dendritic cell activation, immune checkpoint blockade, therapeutic cancer vaccines, oncolytic virus therapy, vascular normalization, and combination therapies involving chemotherapy or radiotherapy with immune-stimulating agents (128).

### 4.1 Physical barriers to immune infiltration

Physical barriers in the TIME suppress immune responses by limiting immune cell access through poor vascularization, lack of T cell homing signals, ECM density, and cellular blockades. Tumor-altered ECM, enriched with collagen and fibronectin, becomes stiffer and more fibrotic, further hindering immune cell infiltration (170). CAFs in the TIME stroma promote tumor growth by secreting growth factors, cytokines, and chemokines, and by remodeling the ECM. Tumor-induced activation of CAFs and inflammation increases interstitial pressure and collagen deposition, leading to ECM stiffening and disorganization through elevated collagen-processing enzymes such as lysyl oxidases (127, 171). Tumors also express high levels of matrix metalloproteinases (MMPs) compared to non-cancerous epithelial cells, which can lead to increased ECM remodeling (172). Tumor cells influence the TIME by using CAFs by recruiting and activating them through paracrine signals such as TGFα, TGFβ, platelet-derived growth factor (PDGF), epidermal growth factor (EGF), and fibroblast growth factor 2 (FGF-2) (173, 174). Activated CAFs reorganize the TIME to promote metastasis, therapy resistance, dormancy, and reactivation. They contribute to the formation of physiological barriers by secreting both cellular and acellular components, thereby impeding drug penetration and treatment efficacy (175) In preclinical mouse models, the TIME has shown a decrease in the monitoring effectiveness of drug delivery and treatment (176).

### 4.2 Reprogramming the immunosuppressive microenvironment

Healthy tissues maintain balanced cytokine levels and lack the hypoxia and acidosis found in the TIME, allowing immune cells to function without metabolic stress (177, 178). Unlike tumors, healthy tissues show a normal distribution of immune cells rather than an accumulation of immunosuppressive cells such as Tregs and MDSCs (179). The TIME contributes significantly to therapeutic resistance, making it a key target for enhancing immunotherapy. Among its key players, Tregs and MDSCs are major contributors to immune suppression. Tregs are characterized by the expression of FOXP3 and CD25, and they suppress effector T cell responses through the secretion of IL-10 and TGF-β, which inhibit T cell proliferation and function (179) Tregs are also highly expressed immune checkpoint molecules that express CTLA-4, PD-1, LAG-3, and TIM-3, and suppress T cell activation through inhibition of DC maturation and co-stimulation (180). Additionally, Tregs disrupt metabolic pathways by expressing CD39 and CD73. This process involves CD39 sequentially breaking down extracellular ATP into ADP and then AMP, while CD73 converts AMP into adenosine, creating an immunosuppressive environment that dampens effector T cell activity (181). Recent studies show activated CD8 T cells can produce adenosine via CD73-containing extracellular vesicles, revealing more complex adenosine pathway regulation than previously understood (182). MDSCs help sustain an immunosuppressive TIME by inhibiting T cells via arginase-1, nitric oxide, and ROS, promoting Treg expansion, and disrupting T cell trafficking through chemokine modulation (176). MDSCs suppress T cells by secreting IL-10, TGF-β, and checkpoint molecules such as PD-L1 and CTLA-4. They expand in response to inflammatory and tumor-derived signals and are divided into two main subtypes: polymorphonuclear (PMN-MDSCs) and monocytic (M-MDSCs), each with distinct mechanisms of action (32). The upregulation of PD-L1 in tumor cells is a hallmark of cancer progression and reflects oncogene-like behavior. This not only enables immune evasion but also supports more aggressive tumor growth and spread (183). Oncogene-driven pathways contribute to this by increasing PD-L1 expression through enhanced transcription, protein stabilization, and gene amplification. These changes help tumor cells avoid immune detection by elevating PD-L1 on their surface, thereby suppressing T cell responses (184).

### 4.3 Modulation of immune checkpoint suppression

Immune checkpoints serve as critical regulatory mechanisms for preventing excessive immune activation and autoimmunity (176). The PD-1/PD-L1 and CTLA-4 pathways are among the most studied immune checkpoints responsible for suppression of T cell activation within the TIME. However, tumors frequently exploit these pathways to suppress CTLs function and evade immune surveillance, thereby promoting tumor progression (185, 186) This PD-L1 binds to PD-1 on CTLs, inducing T cell exhaustion and impairing effector function (186). Similarly, CTLA-4, expressed on T cells, competes with the co-stimulatory receptor, CD28 for binding to CD80/CD86 on APCs, dampening early T cell activation and promoting immune tolerance (187). The TIME further amplifies checkpoint-mediated immunosuppression through metabolic reprogramming, for example, by inducing hypoxia-driven PD-L1 upregulation and recruiting immunosuppressive cell populations (188).

## 5 Stimulation of the immune system with immunotherapy therapy

The activation of the host immune system is crucial for effective targeting tumors, improving outcomes, and reducing treatment side effects (20). In cancer immunotherapy, various approaches have been developed to harness the patient’s immune system to recognize and eliminate tumor cells. Some strategies involve extracting and modifying immune cells ex vivo, such as in CAR T cell therapy, to enhance their tumor-killing capacity (189) Other therapies rely on engineered antibodies to target tumor cells, or to stimulate the immune system to recognize and attack tumor cells using other modalities such as vaccines, cytokine or immune checkpoint inhibitors (190–192). Immunotherapies vary by cancer type and patient factors. This section reviews key treatments: monoclonal antibodies, immune checkpoint inhibitors, bispecific T cell engagers, CAR T cells, and cancer vaccines, focusing on their mechanisms and clinical studies (193).

### 5.1 Monoclonal antibodies

Monoclonal antibodies (mAbs) are immunoglobulins with a high level of specificity (mono-specificity) for a particular antigen or epitope of tumor interest. The mAbs bind to specific antigens or proteins on cell surfaces such as cancer cells and send a signal to the host immune system to destroy tumor cells (190, 194). Upon binding to their targets, mAbs can engage immune effector functions through their Fc region by activating Fc receptors on immune cells such as NK cells and macrophages. This leads to multiple cytotoxic mechanisms, including complement-dependent cytotoxicity (CDC), antibody-dependent cellular phagocytosis (ADCP), and antibody-dependent cellular cytotoxicity (ADCC) (195). MAbs are consist of different classes or origin, using murine, chimeric, humanized or fully human as well as varying in each function being naked conjugate or bispecific which are all dependent on the target (195), see **Table 3**. Fully human monoclonal antibodies targeting chitinase-3-like-1 (CHI3L1) have shown promise in suppressing tumor growth, fibrosis, angiogenesis, and immune remodeling across various cancers. CHI3L1 contributes to an immunosuppressive tumor microenvironment, supporting cancer progression and highlighting its potential as a therapeutic target (196). Su et al., 2024 developed fully human CHI3L1-neutralizing monoclonal antibodies (nAbs) were developed using phage display technology. These nAbs demonstrated high specificity and affinity for CHI3L1 across multiple cancer cell lines, including lung, pancreatic, and colorectal cancers. Functionally, the antibodies reduced tumor-promoting signals, inhibited cancer cell proliferation and migration, and showed potential as therapeutic agents in an immunosuppressive TIME (196).

**Table 3:** Approved monoclonal antibodies (mAbs).

Drug	Target	Type	Approved indications	Year
Brentuximab vedotin (Adcetris)	CD30	Chimeric IgG1 mAb (ADC)	HL, systemic ALCL	2011
Blinatumomab (Blincyto)	CD19	Bispecific T-cell engager (BITE)	B-cell precursor ALL	2014
Elotuzumab (Empliciti)	SLAMF7	Humanized IgG1 mAb	MM (in combination with lenalidomide and dexamethasone)	2015
Daratumumab (Darzalex)	CD38	Human IgG1k mAb	MM	2015
Gemtuzumab ozogamicin (Mylotarg)	CD33	Humanized IgG4 mAb (ADC)	AML	2017
Inotuzumab ozogamicin (Besponsa)	CD22	Humanized IgG4 mAb (ADC)	r/r B-cell precursor ALL	2017
Isatuximab (Sarclisa)	CD38	Human IgG1 mAb	r/r MM	2020
Mosunetuzumab (Lunsumio)	CD20	Bispecific T-cell engager (BITE)	r/r FL	2022
Teclistamab (Tecvayli)	BCMA	Bispecific T-cell engager (BITE)	r/r MM	2022
Epcoritamab (Epkinly)	CD20	Bispecific T-cell engager (BITE)	r/r DLBCL	2023

ADC, antibody-drug conjugate; ALCL, anaplastic large cell lymphoma; ALL, acute lymphoblastic leukemia; AML, acute myeloid leukemia; BCMA, B-cell maturation antigen; DLBCL, diffuse large B-cell lymphoma; FL, follicular lymphoma; HL, Hodgkin lymphoma; MM, multiple myeloma; r/r, relapsed/refractory.

### 5.2 Immune checkpoint inhibitors

The TIME supports tumor growth by recruiting immune and stromal cells that supply nutrients to heterogeneous tumor cell populations. Specifically, T-cell activation targets threats, while Tregs suppress harmful immune responses (91). T cells express checkpoint receptors such as PD-1 and CTLA-4, which are targeted by FDA-approved immune checkpoint inhibitors (ICIs) including anti-CTLA-4, anti-PD-1, and anti-PD-L1 antibodies. These therapies harness cytotoxic CD8^+^ T cells to induce durable anti-tumor responses and long-lasting remissions (91, 197). These immunotherapies block the interaction between immune cell proteins and their partners, called ‘checkpoints,’ which act as brakes on the immune system. ICIs allow the adaptive immune system to respond to tumors more effectively, creating a better treatment for TIME (198, 199). By inhibiting these checkpoints, ICIs enhance the ability of host T cells to more effectively recognize and attack cancer cells within the TIME (200).

Relatlimab is a LAG-3-blocking antibody, a lymphocyte-activation gene 3, and an FDA-approved ICI, see **Table 2** for other ICIs. Relatlimab is a third checkpoint inhibitor shown to reduce T cell exhaustion and enhance anti-tumor activity when combined with nivolumab in previously treated melanoma patients (RELATIVITY-047, NCT03470922). ICI are also known for working well as combination therapy, specifically targeting LAG-3 and PD-1 shows greater progression-free survival than PD-1 inhibition alone in untreated metastatic or unresectable melanoma (201). LAG-3 is not cancer specific, although it has been shown to be associated with aggressive tumor progression (202, 203). LAG-3 is a promising immune checkpoint target; its inhibition can enhance anti-tumor immune responses and potentially overcome resistance to existing therapies. In the CheckMate 040 clinical trial, combining dual ICIs (Ipilimumab and Nivolumab) with the chemotherapy drug Sorafenib showed improved therapeutic efficacy in patients with advanced hepatocellular carcinoma. The combination of Ipilimumab (anti-CTLA-4) and Nivolumab (anti-PD-1), administered after prior treatment with Sorafenib, resulted in the most robust responses and the longest median overall survival (204).

### 5.3 Bispecific T cell engagers

Bispecific T cell engagers (BiTE) are engineered molecules of two single-chain variable fragments (scFv) linked by a flexible connector. Unlike the traditional antibodies that bind to a single antigen, BiTEs are designed to simultaneously target tumor-specific antigens on tumor cells and CD3 on T cells. Clinical-approved BiTEs target CD19 x CD3, BCMA x CD3, or CD20 x CD3 to promote cytotoxicity of T cells. One pair of the targets for BiTEs is scFv, a tumor-associated antigen (TAA) on the tumor cell and CD3 molecule on the T cell (205, 206). Since immune checkpoints and other immunosuppressive factors within the TIME result in a population of anergic T-cells, preventing their redirection to tumor killing and thereby limiting the effectiveness of BiTE therapy (206, 207). Blinatumomab is the first BiTE therapy that has shown to be an effective and long-lasting immunotherapy, blustering the host immune system by taking advantage of the flexibility of targeting multiple antigens simultaneously and potentially being used in a combination therapy (208). Using therapy constructs CD20 × CD3, showed a 37% overall response rate and 19% complete response rate in aggressive non-Hodgkin lymphoma, including CAR T–resistant or relapsed patients, demonstrating activity across multiple treatment lines. The successful clinical trial (NCT02500407) led to the FDA approval of mosunetuzumab-axgb (Lunsumio), see **Table 2** for other BiTE therapies. BiTE-induced T-cell activation leads to cytokine release within the immune synapse, which diffuses to nearby cells and upregulates surface molecules, enhancing anti-tumor activity through a ‘bystander effect (209). Despite their therapeutic potential, BiTEs can trigger cytokine storms, leading to cytokine release syndrome (CRS)—a potentially life-threatening condition marked by excessive cytokine release into the bloodstream (207, 210). Resistance to this immunotherapy has been associated with antigen loss and immunosuppressive factors within the TIME, including upregulated immune checkpoints and enhanced host immunosuppression via membrane trafficking (211, 212). BiTEs have a short half-life due to the engineered structure lacks the Fc region, preventing FcRn recycling. This leads to rapid clearance from circulation and necessitates continuous treatment to maintain therapeutic effectiveness (213, 214).

### 5.4 Chimeric antigen receptor T cell

Chimeric antigen receptor (CAR T) cell therapy is a personalized treatment that modifies patient T cells to help fight cancer. CAR Ts are synthetic receptors that redirect lymphocytes and primarily T cells, to recognize and eliminate cells expressing a specific target antigen (215). Traditionally, antigen-binding domains are constructed from the variable heavy (VH) and light (VL) chains of monoclonal antibodies, which are connected by a flexible peptide linker to form a single-chain variable fragment (scFv). CAR Ts binding to antigens on the cell surface occurs independently of the MHC receptor, resulting in robust T cell activation and potent anti-tumor responses. CAR Ts target the extracellular surface of cancer antigens using MHC-independent T cell activation (216). CAR T cell therapies have received FDA approval, see **Table 4**. Specifically CAR T cell therapy has been used for improving and treating progression-free survival in multiple myeloma, enhancing overall survival in large B-cell lymphoma, and achieving high remission rates in other hematologic cancers, including acute lymphoblastic leukemia, follicular lymphoma, and mantle cell lymphoma (217). Tisagenlecleucel an anti-CD19 CAR T cell therapy produced high rates of complete remission achieved complete response (CR) rates of 71–81% in multicenter clinical trials involving patients with relapsed or refractory B cell acute lymphoblastic leukemia (R/R B-ALL), and a group with limited treatment options (218, 219).

**Table 4:** Approved and emerging CAR T-cell therapies.

Approved CAR T-Cell Therapies				
Drug	Target	Approved indications		Year
Tisagenlecleucel (Kymriah)	CD19	- B-cell B-ALL - r/r LBCL - r/r FL		2017
Axicabtagene ciloleucel (Yescarta)	CD19	- r/r LBCL - r/r FL		2017
Brexucabtagene autoleucel (Tecartus)	CD19	- r/r MCL - r/r B-ALL		2020
Lisocabtagene maraleucel (Breyanzi)	CD19	- r/rLBCL - r/r CLL/SLL		2021
Idecabtagene vicleucel (Abecma)	BCMA	- r/r multiple myeloma (MM)		2021
Ciltacabtagene autoleucel (Carvykti)	BCMA	- r/r MM		2022
Equecabtagene autoleucel (Fucaso)	BCMA	-r/r MM		2023 (China)
Afamitresgene autoleucel = afami-cel (Tecelra)	CD30	-Synovial sarcoma		2024
obecabtagene autoleucel (Aucatzyl)	CD19	-B-cell ALL		2024
Emerging CAR T-Cell therapies				
Drug/Sponsor	Type/Target	Indication	P	Reference
BMS-986393	GPRC5D	r/r and Lenalidomide-refractory MM	3	NCT06615479
TanCAR19/20-T CAR-20/19-T IMPT-314 MBCART2019.1	CD19 & CD20	r/r NHL r/r B-cell malignancies r/r Aggressive B-cell NHL r/r aggressive CD20+ CD19+ B-NHL/CLL/SLL. r/r DLBCL	1/2	NCT03097770 NCT04186520 NCT05826535 NCT03870945 NCT04792489
PBCAR20A	CD20	r/r B-cell NHL or r/r CLL/SLL.	1/2	NCT04030195
bbT369	CD79a and CD20	r/r B Cell NHL	1/2	NCT05169489
CAR20(NAP)-T	CD20 (secrete NAP)	B-cell malignancies.	1/2	NCT06002659
HER2-CAR T	HER2	Metastatic rhabdomyosarcoma.	1	NCT00902044
PSCA-CAR T	PSCA	Prostate.	1/2	NCT02744287

ALL, acute lymphoblastic leukemia; BCMA, B-cell maturation antigen; CLL, chronic lymphocytic leukemia; FL, follicular lymphoma; LBCL, large B-cell lymphoma; MCL, mantle cell lymphoma; MM, Multiple myeloma; NAP, neutrophil-activating protein; NHL, non-Hodgkin lymphoma; P, phase; PSCA, prostate stem cell antigen; r/r, relapsed/refractory; SLL, small lymphocytic lymphoma.

CAR Ts can have cytokine-related toxicities due to host immune system recognition resulting in the implementation of human or humanized antibody fragments instead of the classical murine-derived CARs to lessen CAR T treatment immunogenicity (215, 220). CAR T therapy faces key challenges in treating solid tumors and blood cancers, including toxic off-target effects, modest anti-tumor efficacy, antigen loss by tumors, and an immunosuppressive TIME that blocks tumor clearance (215, 220). CAR T cell therapy can fail due to changes in tumor-associated antigens (TAAs), such as antigen loss or downregulation, which make cancer cells undetectable to CAR T cells (221). Additionally, poor trafficking and infiltration limit CAR T cell access to tumors, reducing treatment effectiveness. Limited CAR T cell expansion and short-term persistence, often caused by exhaustion from co-inhibitory pathways—also contribute to poor responses, posing a challenge for developing longer-lasting CAR T therapies (13).

### 5.5 Cancer vaccines

Cancer vaccines represent a vital component of immunotherapy, designed to either prevent or treat cancer using diverse platforms like cells, viruses, peptides, or nucleic acids, see **Table 5** (222). The goal of cancer vaccines is to train the host immune system to recognize and attack tumor-associated and tumor cells by exposing them to specific cancer-associated molecules such as, antigens, of the tumor of interest (223). Some of the current cancer vaccines are targeting HPV- related cancers, melanoma and prostate cancer (224, 225), as well as other therapies under development for other cancers such as NSCLC, breast cancer and ovarian cancer (224–227). Each specificized cancer vaccine has different success rates, although, HPV-related vaccines are seen as highly effective, while other cancers vary the response rate and are often below 10% (228, 229).

**Table 5:** FDA-approved cancer vaccines.

Drug	Target	Type	Approved indications	Year
Bacillus Calmette Guérin (BCG) (Also TheraCys, branded form of BCG)	Urothelial carcinoma cells	Live attenuated bacterial	Treatment of early-stage non–muscle-invasive bladder cancer (NMIBC)	1990
HBV vaccine (Engerix-B, Recombivax HB, Heplisav-B, PreHevbrio)	Hepatitis B surface antigen (HBsAg)	Recombinant protein	Prevents hepatitis B virus and liver cancer	1989 onwards
Gardasil	HPV types 16, 18, 6, and 11	Virus-like particles	Prevents cervical cancer, anal cancer, vulvar, vaginal, and penile cancers	2006
Cervarix	HPV types 16 and 18	Virus-like particles	Prevents cervical cancers	2009
Sipuleucel-T (Provenge)	Prostatic acid phosphatase (PAP)	Autologous cellular	Treatment of asymptomatic or minimally symptomatic metastatic castration-resistant prostate cancer	2010
Gardasil-9	HPV types 6,11, 16, 18, 31, 33, 45, 52, and 58	Virus-like particles of 16, 18, 31, 33, 45, 52, and 58 proteins	Prevents cervical cancer, anal cancer, vulvar, vaginal, and penile cancers	2014
IMLYGIC (talimogene laherparepvec)	Herpes simplex virus (HSV-1)	Oncolytic virus	Treatment of unresectable melanoma (cutaneous, subcutaneous, and nodal lesions)	2015

HPV, human papillomavirus.

DC vaccines are loaded with tumor-specific proteins and idiotypes, which target and treat specific cancers. Idiotypes produced by tumor cells help stimulate an immune response from the stimulated DC-based vaccine, which is then aimed at attacking the TIME (230). A study using idiotype protein-pulsed DC vaccines for multiple myeloma showed minimal side effects and boosted host immune responses, with patients exhibiting ID-specific immunity, indicating potential anti-myeloma effects (231). Unfortunately, DC-based vaccines have not achieved the strong clinical results initially expected, despite their promise in enhancing anti-tumor immune responses. However, recent studies suggest that combining DC vaccines with ICI, such as CTLA-4 and PD-1 blockers, may improve T cell responses and lead to better clinical outcomes (232–234).

Peptide-based vaccines stimulate T and B cell immunity by targeting specific epitopes, offering high specificity, improved efficacy, and fewer side effects compared to conventional cancer therapies (235, 236). Peptide-based vaccines are known to treat many cancer types such as, melanoma, lung cancer, breast cancer, pancreatic cancer, and even some brain tumors as FDA approved vaccines and vaccines in clinical trial (237–244). These vaccines offer benefits such as stability, safety, and the ability to elicit robust T cell responses, which enable direct monitoring of immune activity and support repeated booster doses (245, 246). An additional study using a HER-2/neu-derived peptide in combination with a linker peptides and Pan HLA-DR epitopes showed enhanced CD4+ and CD8+ T cell responses as well as a replicative result of increased IFN-γ production (247).

Virus-like particle vaccines, such as the widely used HPV vaccine, help prevent genital warts, respiratory papillomatosis, and cancers including cervical, anal, penile, vulvar, vaginal, and oropharyngeal (248, 249). Clinical trials show that adjuvant HPV vaccination significantly reduces the recurrence of CIN 1+ and CIN 2+ after surgical treatment (250). Repurposed antiviral subunit and mRNA vaccines are being studied to reshape the TIME by inducing cancer cell death, releasing tumor antigens, and enhancing immune cell activation for stronger anti-tumor responses (251).

Nucleic acid vaccines (NAVs) use cancer cell DNA and RNA to stimulate the immune system to target cancer cells and the tumor microenvironment by promoting tumor antigen production (252). DNA vaccines enter the nucleus to produce multiple mRNA copies, boosting antigen levels but risking delays and insertional mutations. mRNA vaccines act faster by translating in the cytoplasm without genome integration risk, though they typically yield fewer antigens (222). The Phase I Lipo-MERIT trial (NCT02410733) of the melanoma vaccine FixVac (BNT111) showed a favorable safety profile (225). Targeting four shared tumor-associated antigens, FixVac—alone or with PD-1 inhibitors—induced durable responses and strong CD4+/CD8+ T-cell immunity in advanced melanoma patients previously treated with ICIs, suggesting its promise as a potent RNA-based immunotherapy (225).

## 6 Incorporation of AI into TIME monitoring for cancer therapy

Recent advances in cancer therapy have seen AI become directly embedded within cutting edge technologies, from imaging to molecular analysis. AI-powered tools, such as deep learning algorithms integrated into MRI, PET/CT, and mass cytometry imaging, now enable precise detection of tumor-immune interactions, automated identification and quantification of immune cell populations, and improved prediction of treatment outcomes (253, 254). Additionally, machine learning models are driving molecular data interpretation allowing for more rapid analysis of gene mutations, immune marker profiles, and recommendation of optimal personalized therapies for patients (255). Understanding the complex, heterogeneous TIME is crucial for improving cancer diagnostics and treatment, but its spatial and temporal variability challenges characterization and clinical use (15, 256, 257). To address these challenges, recent advancements have integrated cutting-edge imaging technologies, molecular assays, and computational tools, offering a more comprehensive and dynamic assessment of tumor-immune interactions (258). Imaging modalities have played a pivotal role in showing the TIME’s spatial organization and functional dynamics. Fluorescence and bioluminescence imaging enable real-time tracking of immune cell infiltration, spatial distribution, and dynamic change in preclinical models (259–262). Multiplex immunohistochemistry (IHC) and imaging mass cytometry provide high-resolution spatial mapping of protein expression, allowing for precise characterization of immune cell phenotypes and their interactions with cancer cells (248, 263, 264). Multiplex ion beam imaging enables simultaneous detection of multiple biomarkers at subcellular resolution, revealing immune cell heterogeneity and spatial relationships across tumors (265, 266). A commonly used diagnostic scan in the medical field is the positron emission tomography-computed tomography (PET/CT) scan, which combines the PET and CT scans. A PET/CT scan can observe and record detailed body images to diagnose and treat diseases (267). This imaging technique is used to observe CAR T-cell treatment and many other cancerous mass treatments. Using PET/CT can also monitor tissue metabolism which helps provide more inclusive observations of the TIME (268, 269). Intravital microscopy (IVM) is an imaging technique that maps tumor-associated vessels, measures vessel density, and uses vital dyes to locate vessels within tumors (270). IVM has been used in clinical oncology to characterize superficial vessels in human melanoma tumors and to analyze tumor physiology, drug delivery, and immune cell trafficking (271). Aside from diagnostic imaging, MRI has recently been applied to treatment plans for individual cases. MRI uses strong magnetic fields to align protons in the body and measures their interactions, which are computationally processed to produce high-resolution anatomical images. Recently, MRI has been combined with nanoparticles to improve tumor detection, quantify tumor burden, and track the localization and accumulation of therapeutic agents (272). Additionally, photoacoustic imaging has emerged as a powerful, non-invasive technique, bridging optical and ultrasound imaging to provide high-contrast, deep-tissue visualization of tumor vascularization, metabolic activity, and immune cell infiltration (273, 274). Given the TIME’s impact on current cancer therapies, there is a need for more effective screening, prevention, and treatment strategies to benefit patients.

Beyond imaging, molecular techniques like enzyme-linked immunosorbent assay, quantitative polymerase chain reaction, flow cytometry (FC), and IHC are vital for measuring immune markers and tumor-related biomolecules, enabling assessment of immune activation and therapy-induced changes in the TIME. AI integration further enhances accuracy, efficiency, and prediction in oncology diagnostics and treatment.

(143) Machine learning algorithms are increasingly employed for automated image analysis, enabling rapid identification of tumor-infiltrating lymphocytes, classification of histopathological features, and prediction of patient prognosis based on multi-modal data (70, 275, 276). AI-driven computational models also support treatment decision-making by analyzing vast datasets from genomic, transcriptomic, and proteomic studies, thereby identifying potential therapeutic targets and optimizing personalized medicine strategies (277).

### 6.1 AI-driven insights from biomarkers and liquid biopsies

Liquid biopsies and circulating tumor DNA (ctDNA) from blood samples offer a non-invasive way to assess cancer, monitor treatment response, and detect recurrence earlier than traditional imaging. In non-small cell lung cancer, biomarkers were used to predict immunotherapy outcomes independently of PD-L1 status (278). Biomarkers have become a great tool for screening and monitoring various cancer types and diseases by enabling clinicians to detect cancers at earlier stages, assess the effectiveness of therapies, and adjust treatment strategies based on individual patient responses (279). Many clinical trials have utilized predictive approaches using biomarkers like microsatellite instability (MSI), PD-L1, and tumor mutational burden (TMB) to predict patient response to ICI treatment of tumor types (280, 281). Combining biomarkers in panels offers the advantage of cross-confirmation and can detect changes independent of upregulated pathways, unlike single markers (256). Combining biomarkers like Alpha-Fetoprotein (AFP), a biomarker used to detect liver cancer (hepatocellular carcinoma) and germ cell tumors in the testicles or ovaries with cfDNA improving diagnostic accuracy and variety (282). Additional studies have found that combining different panels of biomarkers for breast cancer diagnosis improved performance over using cancer antigen 15-3 (CA15-3) or carcinoembryonic antigen (CEA) alone (30). Biomarkers detect gene mutations and tumor DNA to predict treatment response and safety, mainly identifying patients likely to benefit from immunotherapy. However, not all patients receive biomarker-based treatments due to tumor-specific factors (283–285).

**Figure 4:**
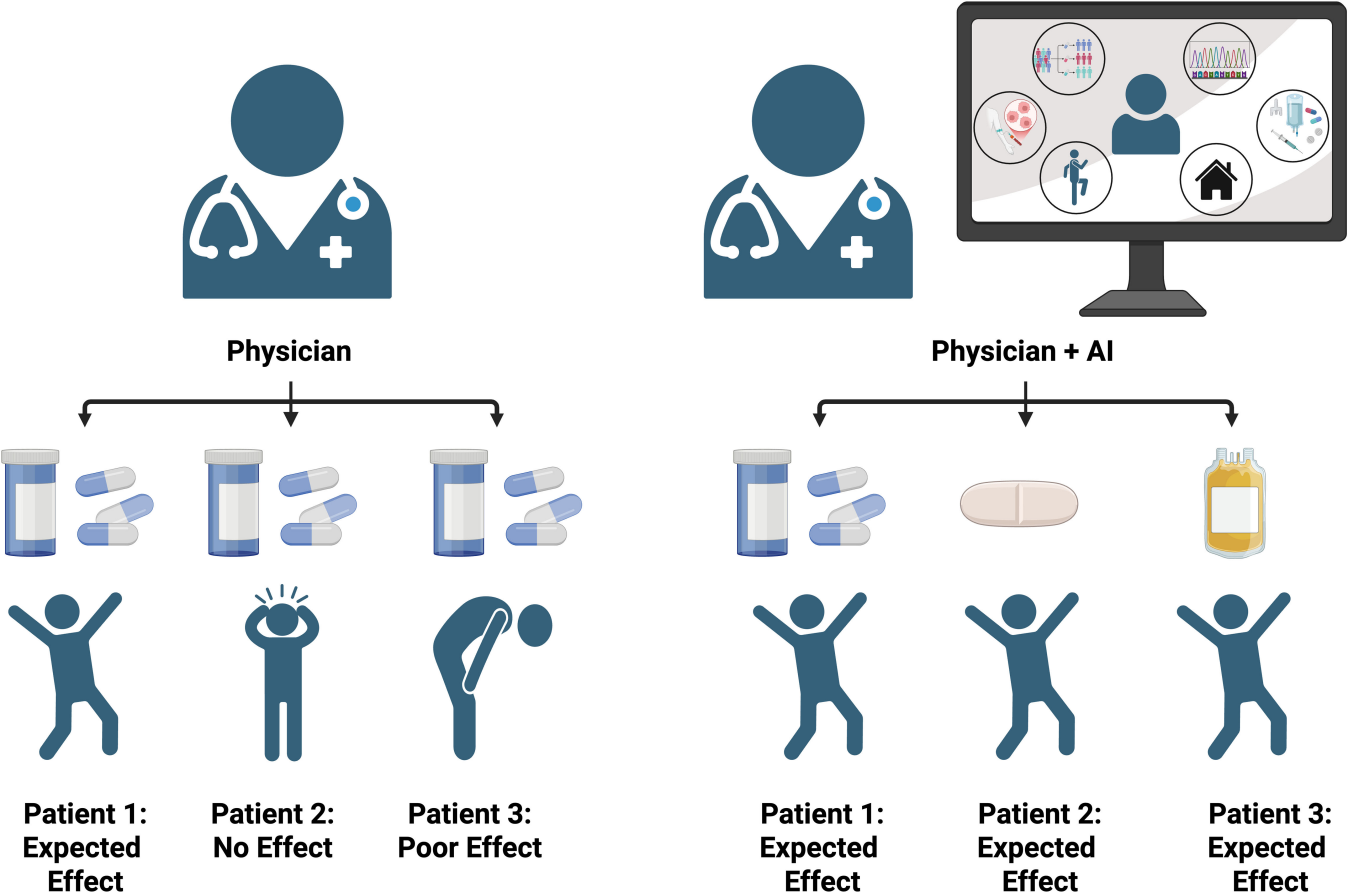
Comparison of patient outcomes with physician-only care versus integrated physician and AI-guided treatment. The AI-assisted approach leverages biomarkers, sequencing data, environmental factors, and treatment histories to personalize therapy, resulting in more consistent and optimized patient outcomes compared to physician evaluation alone.

The incorporation of AI models has successfully predicted patient survival and response to therapies by analyzing patient TIME and clinical data. AI empowers oncologists to develop personalized treatment plans tailored to each patient’s genetic and molecular profile, **Figure 4**. This approach enhances the likelihood of successful treatment outcomes while reducing the risk of adverse side effects when treating the TIME (286). Integrating AI into monitoring and observing cancer progression has contributed significantly to the ongoing progress in biomedical cancer research, driving innovation and improvement in cancer treatment (287). The development of PERCEPTION (PERsonalized Single-Cell Expression-Based Planning for Treatments In ONcology), a precision oncology computational pipeline, has produced promising results for prediction and diagnosis. This pipeline is based on publicly available matched bulk and single-cell expression profiles from large-scale cell-line drug screens from patients’ single-cell tumor transcriptomics. The goal of PERCEPTION is to predict responses to targeted therapies in cultured and patient-tumor-derived primary cells from two clinical trials for multiple myeloma and breast cancer patients and the development of resistance to kinase inhibitors against lung cancer patients. The single-cell expression profiles have showcased patient stratification using tumor profiles and the available oncology tools to predict patient response and resistance to treatments (288). Biomarkers aid in detection and outcome management, though their clinical use can be complex. Integrating AI with biomarker data, particularly in liver cancer, shows promise in research and potential clinical applications. Studies have shown that using biomarkers using a predictive model for identifying the risk of hepatocellular carcinoma (HCC) one year in advance achieved an area under the receiver operating characteristic curve (AUROC) of 0.94, with a 95% confidence interval of 0.937 to 0.943. This model demonstrated a sensitivity of 0.869 and a specificity of 0.865. For predicting HCC at different time points, the AUROC values were as follows: 0.96 for 7 days, 0.94 for 6 months, 0.94 for 1 year, 0.91 for 2 years, and 0.91 for 3 years in advance (280, 289). Machine learning was used to develop a predictive model for diagnosing HCC, optimized with grid search to select the best hyperparameters. Trained on data from 539 HCC and 1,043 non-HCC patients, the gradient boosting model achieved the highest accuracy (87.34%) and an AUC of 0.940. Compared to single tumor markers, this approach significantly reduced misclassification, demonstrating the value of biomarker-based ML models (290, 291). AI and machine learning enable advanced models for cancer diagnosis and sub-classification using histopathology images, offering improved accuracy by handling population diversity and slide variability, enhancing patient sample analysis.

### 6.2 Enhanced tissue analysis: transforming immunohistochemistry and cancer evaluation

IHC is a method that uses tissue samples from patients from biopsy and processed where specific antibodies are selected to identify the presence of the target antigen (292). IHC slides are read by pathologists by analyzing the patient tissue samples by assessing cellular morphology, identifying cancer cells, evaluating treatment responses, and detecting specific cancer markers for tumor recognition and progression (293). AI and machine learning are used alongside pathologists to identify potential patterns and cancerous morphology in patient samples. These technologies help uncover additional findings, enhancing the accuracy of cancer cell identification and diagnosis. This collaborative approach is increasingly being implemented in cancer research and clinical settings. This technology is called the Clinical Histopathology Imaging Evaluation Foundation (CHIEF) model, a general-purpose weakly supervised machine learning framework to extract pathology imaging features for systematic cancer evaluation. This technology uses two pretraining methods to extract diverse pathology representations from sample slides: unsupervised pretraining for tile-level identification and weakly supervised pretraining for whole–slide pattern recognition during training sessions. CHIEF was trained using 60,530 whole slide images using 16 different anatomical sites (294). This research underscores AI’s significant potential to enhance the accuracy and reliability of biomarker detection, particularly in the context of liver cancer diagnosis and treatment.

Immune modulation has also become a focal point for AI integration due to the pivotal role immune therapy can provide for patients by using their immune system. AI is utilized to reveal underlying immune patterns associated with treatment responses, indirectly and directly; this approach predicts how patients will respond to immunotherapy. Including AI in analyzing high-throughput genetic sequences and medical imaging provides crucial insights for managing cancer immunotherapy. This analysis aids in selecting suitable patients, optimizing treatment strategies, and predicting personalized prognoses (295, 296).

### 6.3 Single-cell based analysis incorporating AI

FC is a widely adopted single-cell-based assay extensively used in cancer diagnostics. FC enables quantitative analysis of immune cells, tumor characterization, and treatment response assessment. It provides high-resolution insights into cell phenotypes and functions, allowing clinicians to monitor circulating tumor cells, cancer stem cells, tumor antigens, and immune subsets in blood, tissue, or bone marrow over time (297). Traditional methods for analyzing the TIME, such as manual assessment of TILs, are labor-intensive and subject to interobserver variability (298). Recent advancements have introduced AI-powered tools that enhance the precision and efficiency of TIME evaluation. For instance, an AI-based spatial analysis system has been developed to classify TIME into immune phenotypes: ‘inflamed,’ ‘immune-excluded,’ and ‘immune-desert’, which correlate with responses to immune checkpoint inhibitors in NSCLC patients (299). Additionally, deep learning frameworks like ImmunoAIzer have been utilized to characterize cell distribution and gene mutations within the tumor, facilitating a more comprehensive understanding of tumor-immune interactions (300). These AI-driven approaches enable high-throughput, objective, and reproducible analyses of the TIME, thereby informing treatment strategies and potentially improving patient outcomes.

### 6.4 Limitations of using AI in cancer diagnosis and therapy

Utilizing AI for cancer detection and therapy can provide complex multi-omics data, AI-powered models can uncover novel therapeutic targets and guide more precise, personalized treatment strategies. As AI continues to evolve and integrate into healthcare, its limitations and challenges must be carefully addressed, monitored, and managed to ensure safe and effective use. Although AI has potential in cancer diagnosis and therapy, its use also comes with drawbacks, such as the risk of inaccuracy and hallucinations. These issues often arise from outdated or poorly reviewed data, which can lead to misleading recommendations and suboptimal clinical decisions (301, 302). Data bias is a partially due to inaccuracy die to algorithms being trained in unrepresentative datasets that miss populations of patients, resulting in low accuracy of underrepresented groups, increasing the potential for health disparities in marginalized populations (302, 303). The use of AI in oncology raises concerns about patient privacy and data security, especially when handling sensitive genetic information. Additionally, as clinical practices evolve or data distributions shift, AI algorithms may experience performance degradation over time, leading to inconsistent or unreliable outcomes (302, 304). Health care and research can work to overcome these new and constantly evolving limitations by improving accuracy and reducing errors by stringently reinforcing the hybrid approaches that integrate AI predictions with clinician oversight which often referred to as ‘human-in-the-loop’ models. This approach helps reduce errors and build trust in AI-driven recommendations (305, 306). To reduce bias and reduce health disparities, mitigating bias and promoting equity through training AI algorithms on large, diverse, and representative datasets helps minimize bias and improve outcomes for underrepresented populations (307). Federated learning enables models to learn from data across multiple institutions without sharing raw patient information, thereby enhancing both data diversity and privacy (308, 309). In conclusion, overcoming the limitations of AI in cancer therapy requires a combined approach and leveraging technical solutions such as diverse datasets, explainable models, real-time monitoring, and federated learning, alongside systemic measures including workflow integration, regulatory clarity, data privacy, and cross-disciplinary collaboration—to support ethical, equitable, and effective clinical deployment.

### 6.5 Personalized care for patients

Personalized medicine (PM) refers to a medical approach that tailor’s prevention, diagnosis, and treatment strategies based on an individual’s unique biological characteristics, including genetic, epigenetic, proteomic, and metabolic profiles (310, 311). In oncology, PM uses molecular profiling of the patient and tumor to identify mutations and biomarkers, guiding targeted therapies that maximize efficacy and minimize side effects. For instance, breast cancer patients with HER2 overexpression benefit from HER2-targeted agents such as trastuzumab, while NSCLC patients harboring EGFR mutations or ALK rearrangements are treated with corresponding tyrosine kinase inhibitors. Adoptive Cell Transfer (ACT) therapy, including CAR T and tumor-infiltrating lymphocyte (TIL) therapy, represents a highly personalized approach to cancer treatment using a patient’s immune cells to enhance their anti-tumor response. CAR T-cells therapy involves engineering T cells to express synthetic receptors that recognize specific tumor antigens. In contrast, TIL therapy relies on expanding naturally occurring tumor-infiltrating lymphocytes that have already recognized cancer cells (312, 313). Using a patient’s own immune cells, ACT offers high specificity with minimal rejection risk. It has shown strong efficacy in blood cancers, with CAR T-cell therapies targeting CD19 achieving high remission rates in leukemia and lymphoma (314–316). However, outcomes in solid tumors remain less impressive due to challenges such as tumor heterogeneity, antigen escape, and the immunosuppressive nature of TIME (317–319). Regulatory T cells, MDSCs, and inhibitory cytokines in the TIME can limit ACT by suppressing T-cell function. To enhance efficacy, strategies like combining ACT with immune checkpoint inhibitors help counter immunosuppression and prevent T-cell exhaustion (320). Additionally, approaches targeting the TIME itself, such as depleting immunosuppressive cell populations (321) or modulating the tumor vasculature (322), are being investigated to create a more favorable environment for ACT.

## 7 Discussion

The TIME coordinates cell behavior, shaping the tumor and immune environments both individually and collectively. This review highlights how these microenvironments adapt to evade current immunotherapies and treatments in patients and clinical trials.

Regulating the TIME by balancing activation and suppression is key to improving therapies that boost the host immune response reducing tumor burden and prevent immune evasion (9). Despite ongoing advancements in treatment development, a deeper understanding of the mechanisms by which the TIME adapts to therapies is still needed to overcome treatment resistance and improve therapeutic efficacy. Advances in science and AI have driven personalized cancer therapies by enabling tailored screening and early diagnostics from patient samples (258, 286). Understanding TIME behavior allows researchers and clinicians to combine immunotherapies, AI-driven early detection, and dual treatments to reduce tumor burden and counter immune suppression.

### 7.1 Current challenges caused by the TIME

A key challenge currently being explored by researchers and clinicians is how to monitor the complex and heterogeneous cell populations that comprise the TIME.

The TIME consists of diverse cell types including immune, fibroblasts, epithelial, endothelial and matrix components that interact with tumor vasculature to promote growth and invasion. Variations in cell composition across TIME regions create distinct functional profiles that impact treatment response (15). Tumor heterogeneity includes intratumoral heterogeneity, which refers to the diverse cancer cell populations within a single tumor that can exhibit distinct genetic, molecular, and phenotypic traits. In contrast, intertumoral heterogeneity refers to differences observed across tumors from different individuals, even when diagnosed with the same cancer type (323). The variation within the heterogenous population leads to various treatment responses which can lead to resistance highlighting the difficulties of using standard of care treatments compared to more targeted therapies in PM.

TAAs are the target for specialized CAR T cell therapies used to activate T cells and the destruction of tumor cells (315). Within the heterogeneity of an individual TIME, tumor antigenic heterogeneity will reduce and impair the therapeutic efficiency of adoptive cell therapies, CAR T cell therapy is known for having significant implications of antigen escape occurs within a patient undergoing treatment (324). This escape mechanism can lead to the antigen escape and potential tumor recurrence and immune therapy resistance due to the heterogeneous TIME having the ability to downregulate the targeted antigen of the adoptive cell therapy for treatment (325). Demonstrating that antigen escape driven by TIME heterogeneity has shown to be a predictor of resistance and recurrence, with implications for therapy design and or monitoring (324). A potential tool to overcome this escape mechanism is to target multiple TAAs using CAR T therapy to improve treatment, called 4dem CARs (TanCARs). The development of TanCARs is using a CAR construct with two antigen recognition domains to target two different TAAs simultaneously (326). TanCARs have shown promise in preclinical and clinical studies, demonstrating more durable responses and lower relapse rates compared to monospecific CARs, particularly in cancers prone to antigen escape. Additionally, certain TanCAR designs enhance safety by requiring engagement of both antigens for full activation, increasing specificity and potentially reducing off-target effects such as cytokine release syndrome (326, 327). In a 2020 study, Tong and colleagues developed a series of TanCARs targeting CD19 and CD20, demonstrating that TanCAR7 T cells provide dual antigen coverage and induce a potent, durable anti-tumor response—helping to prevent relapse due to antigen escape following CD19- or CD20-targeted therapies. Notably, no cases of grade 3 or higher CAR T-cell-related encephalopathy syndrome (CRES) were reported during treatment of relapsed/refractory non-Hodgkin lymphoma (r/rNHL) (13, 328). While TanCARs offer enhanced activation through bivalent engagement, this increased signaling strength can sometimes lead to T cell exhaustion, reduced persistence, or heightened toxicity. Their effectiveness also depends on a minimum threshold of antigen expression; if one or both target antigens are present at low levels, the therapeutic response may be diminished. Moreover, in rare cases, tumors may escape treatment by downregulating or losing both antigens, leading to potential resistance even with TanCAR therapy (13, 327). TanCARs are currently in development at various stages within clinical trials. One such trial is NCT07032129, which targets BCMA/GPRC5D in patients with relapsed/refractory multiple myeloma (BAH2573).

TIME can inhibit anti-tumor immune responses by attracting immunosuppressive cells, such as regulatory T cells and myeloid-derived suppressor cells, and by enhancing the expression of inhibitory molecules on immune cells. The TIME can cause tumor-infiltrating T cells to become exhausted, impairing their ability to recognize and destroy cancer cells effectively (21, 127). Additionally, the TIME can increase the expression of immune checkpoint proteins, such as PD-1 and CTLA-4, on immune cells, further suppressing their activity. A study using the incorporation of doxorubicin with nivolumab showed a benefit, metastatic triple-negative breast cancer (TNBC) patients with a 23% objective response rate compared to PD-1/PD-L1 blockade in phase II clinical trial showed no benefit. This study also found that doxorubicin and cisplatin treatment induced the upregulation of genes involved in the T cell cytotoxicity pathway, establishing a link between the clinical activity of these agents and their capacity to regulate systemic immunity (329).

The potential barriers of the TIME have been linked to chemotherapy treatment and metabolic constraints, limiting the effectiveness of treatment and physical barriers created by the TIME (186). Decompression is another method used to reduce physiological pressure, improving oxygenation and reducing drug resistance (330). To overcome T cell exhaustion, strategies include blocking inhibitory receptors (e.g., PD-1, CTLA-4) and using epigenetic, transcriptional, metabolic, and cytokine-based therapies (331).

### 7.2 Future directions

Immunotherapies often work well with other therapies like surgery, chemotherapy, or radiation, which has become an effective treatment due to a simultaneous targeting mechanism that stimulates a host immune response to disrupt the TIME and overcome the immune suppression (332). This approach helps reduce drug resistance while offering multiple anti-cancer benefits, including slowing tumor growth, lowering metastatic potential, targeting mitotically active cells, decreasing cancer stem cell populations, and inducing apoptosis (333). However, the use of combination therapies has become a new line of treatment, due to the clinical benefits in certain cancers that previously had a poor prognosis (334, 335). Combination therapies tend to consist of one or more FDA-approved therapy agents that target similar pathways or mechanisms of cell death as they aim to improve patient outcomes. Combination therapy can reduce treatment costs through full or partial FDA-approved agents and repurposing existing drugs alongside novel therapies shows promise in reducing tumor burden (283). Nivolumab, a well-known immunotherapy and the first PD-1 inhibitor to be paired with classic chemotherapy, demonstrated superior overall and progression-free survival. The clinical trial showed benefits regarding an acceptable safety profile in combination with chemotherapy compared to chemotherapy alone and is now a new standard of first-line treatment for previously untreated patients with advanced gastric, gastro-esophageal junction, or esophageal adenocarcinoma (336).

The incorporation of AI into monitoring approaches for the TIME of cancer patients before, during, and after the treatment duration which can help prevent recurrence of various cancer types by detecting when tumor cells become less responsive to previous therapies in near real time (255). While traditional cancer monitoring remains standard, integrating early relapse detection, adaptive precision medicine, and real-time toxicity prevention can greatly improve outcomes. This requires better data interoperability and machine learning models that ensure privacy, explainability, safety, and accountability. With improved data integration and collaboration, AI can reduce treatment complications and enhance cancer care effectiveness. The use of immunotherapy in cancer treatment is rooted in significant advancements in understanding the key mechanisms behind T-cell activation and suppression. Innovative therapies, such as CAR T cells, CAR NK cells, and CAR M cells, are showing potential in effectively targeting solid tumors (312). The complex TIME poses significant challenges for developing universal cancer therapies and can lead to unintended side effects, such as immune-related adverse events, complicating treatment efforts. Our understanding of the TIME is still incomplete, with many aspects of its biology yet fully explored. These challenges underscore the importance of ongoing research to deepen our knowledge of the TIME and develop safer, more effective therapies.
